# Antimicrobial resistance of clinical bacterial isolates according to the WHO’s AWaRe and the ECDC-MDR classifications: the pattern in Ghana’s Bono East Region

**DOI:** 10.3389/frabi.2023.1291046

**Published:** 2023-12-07

**Authors:** Williams Walana, Ezekiel Kofi Vicar, Eugene Dogkotenge Kuugbee, Francis Sakida, Iddrisu Baba Yabasin, Eric Faakuu, Solomon Amfoabegyi, Juventus Benogle Ziem

**Affiliations:** ^1^Department of Microbiology, School of Medicine, University for Development Studies, Tamale, Ghana; ^2^Department of Clinical Microbiology and Immunology, School of Medical Sciences, C. K. Tedam University of Technology and Applied Sciences (CKT-UTAS), Navrongo, Ghana; ^3^School of Medicine, University for Development Studies, Tamale, Ghana; ^4^Department of Anaesthesiology and Intensive, School of Medicine, University for Development Studies, Tamale, Ghana; ^5^Department of Anatomy, School of Medicine, University for Development Studies, Tamale, Ghana; ^6^Holy Family Hospital, Techiman, Bono-East Region, Ghana

**Keywords:** antimicrobial resistance (AMR), multidrug resistance, MDR, antibiotics, Ghana

## Abstract

**Introduction:**

Antimicrobial resistance (AMR) remains a significant health challenge globally and nations have the responsibility to maintain a constant surveillance of AMR, particularly for the emergence of multidrug-resistant (MDR) isolates to existing antibiotics. Against this backdrop, we applied the WHO’s AWaRe (ACCESS, WATCH, and RESERVE) antibiotics classification and the European Centre for Disease Prevention and Control (ECDC)’s multidrug resistance definition for AMR isolates from clinical specimens.

**Method:**

This study reviewed bacterial culture and antibiotic sensitivity test outcomes. These results were then grouped according to the AWaRe and ECDC-MDR classifications.

**Results:**

In all, the culture and sensitivity results of the 3,178 clinical specimens were investigated, of which 59.5% were from female patients. The pathogens were isolated from 1,187 specimens (37.4%). The WHO’s ACCESS antibiotics, tetracycline, showed a relatively high level of insusceptibility, particularly among Gram-positive (GP) isolates (ranging from 66.7% to 76.7%), along with augmentin (ranging from 44.7% to 81.3%) and cloxacillin (ranging from 50.0% to 78.1%). However, the Gram-negative (GN) isolates showed a relatively high level of susceptibility to amikacin, augmentin, and nitrofurantoin. The WHO’s WATCH antibiotics, cefuroxime, ceftriaxone, cefotaxime, and ciprofloxacin showed a relatively high level of non-responsiveness among the GN isolates, particularly *Proteus* (ranging from 31.4% to 78.4%), *Pseudomonas* (ranging from 21.4% to 96.4%), and *Enterobacter* (ranging from 62.5% to 100%) spp. Among the WHO’s RESERVE antibiotics, resistance to ceftazidime was commonly associated with the GN coliform isolates: *Eschericha coli*, *Klebsiella*, and *Citrobacter* spp. Insusceptibility to meropenem was frequently observed in *Staphylococcus* spp., *E. coli*, coliforms, and *Proteus* spp. Out of the 1,187 isolates, 15.5% (184) were GAT (gentamycin, ampicillin, and tetracycline) MDR, of which 61% (112/184) were from specimens of female patients. The most predominant GAT-MDR isolates were *Staphylococcus* spp., *E. coli*, coliforms, and *Klebsiella* spp.

**Conclusion:**

In conclusion, the study revealed a relatively high level and diverse range of AMR. However, MDR in accordance with the ECDC definition was relatively low. There is, therefore, a need to have further research on AMR to inform national criteria for MDR in Ghana.

## Introduction

Antimicrobial resistance (AMR) is a change in the response of bacteria, viruses, fungi, and parasites to the effects of antimicrobial agents, thus making them unresponsive (World Health, 2021). In bacterial infections, antibiotics serve a good purpose in reducing disease morbidity and mortality, but abuse of these medications has led to varying degrees of antibiotic resistance in most parts of the world (Prestinaci et al., 2015). The resistance occurs naturally following exposure to antibiotic medications. The vulnerable bacteria are killed or suppressed; however, inherently resistant strains remain and continue to proliferate. The resistance is made worse by the wrong dosing of antibiotics, poor adherence to antibiotic treatment regimens, usage of substandard antibiotics, among others (Prestinaci et al., 2015). AMR is still a significant public health concern because it poses a huge threat to preventing and treating the increasing number of bacterial and other microbial infections. Antibiotic resistance, especially, has become a pressing issue as bacteria that cause a variety of infections have become resistant to new antibiotics that enter the market (Prestinaci et al., 2015).

In low- and middle-income countries (LMICs), antibiotics are necessary but sometimes scarce resources. Their utilization is unrestricted, usually abused, and overused (Ayukekbong et al., 2017). Owing to the resistance, there is an increase in disease-related morbidity and mortality. It is particularly problematic when it comes to treating typhoid fever, tuberculosis (TB), and pneumococcal meningitis. It is necessary to improve access to diagnostic laboratories, monitoring of the emergence of resistant strains, and regulation of antibiotic use to maintain the usefulness of antimicrobial drugs in developing countries (Ayukekbong et al., 2017).

AMR, particularly multidrug-resistant (MDR), extensively drug-resistant (XDR), and pan-drug-resistant (PDR) bacteria are of significant concern globally (Van Duin & Paterson, 2016). MDR is defined as acquired resistance to at least one antimicrobial agent in three or more antimicrobial categories, whereas XDR is a resistance to at least one antimicrobial agent in all but two or fewer antimicrobial groups, that is, isolates that are still susceptible to only one or two drug categories, and PDR is defined as the complete resistance to all antimicrobial agents (Magiorakos et al., 2012). MDR bacteria are often linked with nosocomial infections, although some have become common etiologies of community-acquired infections (van Duin and Paterson, 2020). For infections occurring as a result of MDR agents, Gram-negative (GN) microbes account for high mortality rates (Bassetti and Righi, 2013).

The Centers for Disease Control and Prevention (CDC) has classified the MDR bacteria according to their threat levels into urgent threats (pathogens including *Chloridoids difficile*, drug-resistant *Neisseria gonorrhoeae*, carbapenem-resistant *Acinetobacter baumannii*, carbapenem-resistant Enterobacteriaceae, and others) and serious threats [pathogens including drug-resistant *Campylobacter*, vancomycin-resistant *Enterococcus*, carbapenem-resistant *Pseudomonas aeruginosa*, drug-resistant *Salmonella typhi* and non-typhoidal *Salmonella*, methicillin-resistant *Staphylococcus aureus* (MRSA) and others] (van Duin and Paterson, 2020). The European Centre for Disease Prevention and Control (ECDC) defines MDR as non-responsive bacterial isolates to at least one agent in a minimum of three antibiotic categories (Magiorakos et al., 2012). The WHO’s AWaRe classification also separates antibiotics into three groups (i.e., ACCESS, WATCH, and RESERVE) to guide antibiotic prescription (Mudenda et al., 2023).

In Ghana, a study conducted at the Komfo Anokye Teaching Hospital, identified an average multidrug resistance of 89.5%, ranging from 53.8% in *Enterobacter* spp. to 100% in *Acinetobacter* spp. and *P. aeruginosa* (Agyepong et al., 2018). MDR was seen in a combination of ampicillin, tetracycline, chloramphenicol, and cotrimoxazole (Newman et al., 2011). Similar studies identified 41.6% of bacterial isolates to be MDR. In addition, another 49.6% of MDR GN isolates have been reported (Labi et al., 2020; Gnimatin et al., 2022).

In 2012, the WHO published “The Evolving Threat of Antimicrobial Resistance – Options for Action” (World Health, 2012). It outlined a range of interventions that included improving how antibiotics are used in hospitals and the community, preventing and controlling infections, encouraging the manufacture of effective antimicrobials, and having governmental support on antimicrobial stewardship, as well as improving health systems and surveillance on antimicrobial resistance. Antibiotic susceptibility test results of bacterial isolates from clinical samples are collected as part of the surveillance of antibiotic resistance. Such data can be correlated with demographic and clinical information of the patients from whom the samples were taken. These data can then be used to establish and predict the pattern of evolving AMR and emerging MDR (Johnson, 2015). Against this background, this study investigated the pattern of AMR in the Holy Family Hospital in the Bono East Region of Ghana. Furthermore, the study applied the ECDC’s definition of MDR and the WHO’s AWaRE classification to identify the variations in antibiotics whereby no response was obtained.

## Materials and methods

### Ethics approval for the study

The study was officially granted permission by the Institutional Review Board of the University for Development Studies (UDS/RB/032/23). Site permission was obtained from the Medical Director of the Techiman Municipal Health Directorate, the Medical Director of the Holy Family Hospital (HFH), and the Clinical Coordinator of the hospital, as well as the Laboratory In-Charge of the microbiology unit. Data retrieval was done anonymously, and confidentiality was ensured.

### Study design and site

This institutional-based retrospective cross-sectional study was conducted at the microbiology laboratory unit of the HFH in Techiman, in the Bono East Region of Ghana. The hospital is under the Techiman Municipal Health Directorate. With a staff number of about 681 and a total bed capacity of more than 300, the hospital serves as the main referral facility for most hospitals in the Techiman Municipality and the Bono East Region. The hospital was established in 1954 by the Medical Missions Sisters. Initially, under the ownership of the Catholic Diocese of Sunyani in 1977, the hospital was, in 2008, handed over to the Catholic Diocese of Techiman. The hospital is now serving as the municipal hospital for the Techiman Municipality. In the provision of healthcare, the hospital has an accident and emergency unit, an obstetrics and gynecology unit, pediatrics, surgical, and internal medicine units, an ear, nose, and throat (ENT) clinic, a dental clinic, an ophthalmology unit, a general outpatients department, and laboratory, endoscopy, radiology, mental health, physiotherapy, reproductive and child health units, as well as other specialized services. This facility was selected because of its large diversity of patient visits and the variety of samples sent daily to the microbiology unit for bacterial culture and antibiotic sensitivity testing; therefore, giving the hospital a relatively wide population coverage.

### Data collection on clinical specimens and isolate identification

To study the patterns of antimicrobial resistance, a retrospective cross-sectional study was performed at the HFH. The clinical specimens received by the laboratory for culture and sensitivity tests included blood, urine, wound, high vaginal swabs (HVSs), urethral swabs and other swabs, sputum, cerebrospinal fluid, abscesses, and aspirates. The specimens received were recorded into the laboratory register and cultured following standard protocols. Blood agar and MacConkey agar were used routinely for bacterial isolation, whereas Sabouraud glucose agar was used for fungal isolation, particularly for *Candida* species. The culture conditions were set at 37°C aerobically for 18 h to 24 h. The isolate identification was done traditionally using Gram staining, which was followed by biochemical tests such as catalase, coagulase, oxidase, urease, citrate utilization, indole, and triple sugar iron agar. The patients’ demographic properties included age, sex, year of test, and details on specimens. The test outcomes were extracted from the laboratory register. To collect the data, a structured Google (Google Inc., Mountain View, CA, USA)-based questionnaire was generated following the details in the laboratory register. The data available from 2020 to 2022 were extracted via the Google-based form.

### Antibiogram assay by the WHO AWaRe classification

Various antibiotics were used for the sensitivity assay of the isolates. The antibiotics used included 10 from the WHO’s ACCESS group, 10 from the WATCH group, and two from the RESERVE group. The Kirby–Bauer disk diffusion technique on Mueller–Hinton agar was used to test the various antibiotics for their sensitivity or resistance. The ACCESS group antibiotics tested were ampicillin (10 µg), tetracycline (30 µg), trimethoprim/sulfamethoxazole (cotrimoxazole) (25 µg), gentamicin (10 µg), chloramphenicol (10 µg), amikacin (30 µg), amoxicillin/clavulanate (augmentin) (30 µg), nitrofurantoin (300 µg), cloxacillin (25 µg), and penicillin [10 international unit (IU)]. The WATCH group antibiotics evaluated were cefuroxime (30 µg), vancomycin (30 µg), ceftriaxone (30 µg), cefotaxime (30 µg), ciprofloxacin (30 µg), piperacillin (100 µg), erythromycin, (5 μg) norfloxacin (5 μg), nalidixic acid (30 μg), and levofloxacin (5 μg). The RESERVE group antibiotics tested were ceftazidime (30 μg) and meropenem (10 µg).

### Data analysis

The data were extracted from the Google Forms platform, and data entry and validation were done using Microsoft Excel^®^ 2019 (Microsoft Corporation, Redmond, WA, USA). The data analysis was performed with GraphPad Prism 9.0.2 (GraphPad Software Inc., CA, USA) software, and presented as absolute counts, and in proportions, where appropriate. The heatmaps were generated to allow for the easy identification of the antibiotic resistance pattern.

## Results

### Characteristics of the patients

The total number of samples received by the laboratory for analysis from January 2020 to December 2022 was 3,178. The highest number of samples analyzed over the study period was in 2022, with a total sample collection of 1,578 (49.7%). For 2021 and 2020, 31.0% and 19.3% of the total number of specimens were recorded, respectively. Categorizing the number of specimens in terms of sex, 59.3% (1,884) were from females and 40.7% (1,294) from males. The records on the age of patients were available for only 25.7% (819) of patients (see **Table 1**).

**Table 1:** Patients’ demography.

Year	Frequency Number (n)	Percent
2020	616	19.3
2021	984	31.0
2022	1,578	49.7
Total	3,178	100
Sex		
Female	1,884	59.3
Male	1,294	40.7
Total	3,178	100
Age (years)		
≤21	203	24.8
22–39	311	38.0
40–59	168	20.5
≥60	137	16.7
Total	819	100.0

### Frequency distribution of the specimens and isolates

**Figure 1:**
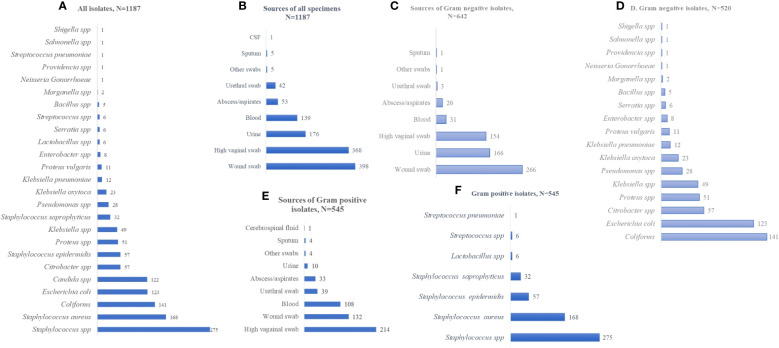
Frequency distribution of specimens and isolates. **(A)** Distribution of all isolates, N=1187. **(B)** Sources of all specimens, N=1187. **(C)** Sources of Gram negative isolates, N=642. **(D)** Gram negative isolates, N=520. **(E)** Sources of Gram positive isolates, N=545. **(F)** Gram positive isolates, N=545. The values displayed are absolute.

Out of the 3,178 specimens received for bacterial culture and sensitivity assay analysis, pathogenic isolates were obtained from 1,187 specimens (37.4%) (**Figure 1A**). Collectively, the most frequently collected samples from which bacterial pathogens were isolated included wound swabs, HVSs, urine, and blood, **Figure 1B**. Stratifying the specimens as sources of either GN or Gram-positive (GP) isolates, GN isolates were often isolated from wound swabs, urine, HVSs, and blood, whereas GP isolates were mostly isolated from HVSs, wound swabs, blood, and urethral swabs (**Figures 1C, E**). The dominant GN bacteria isolated were coliforms, *Escherichia coli*, *Citrobacter* spp., *Proteus* spp., *Klebsiella* spp., and *Pseudomonas* spp. (**Figure 1D**), whereas the GP isolates were predominantly *Staphylococcus* spp., *Lactobacillus* spp., and *Streptococcus* spp. (**Figure 1F**).

### Distribution of isolates by year, sex, and age

**Figure 2:**
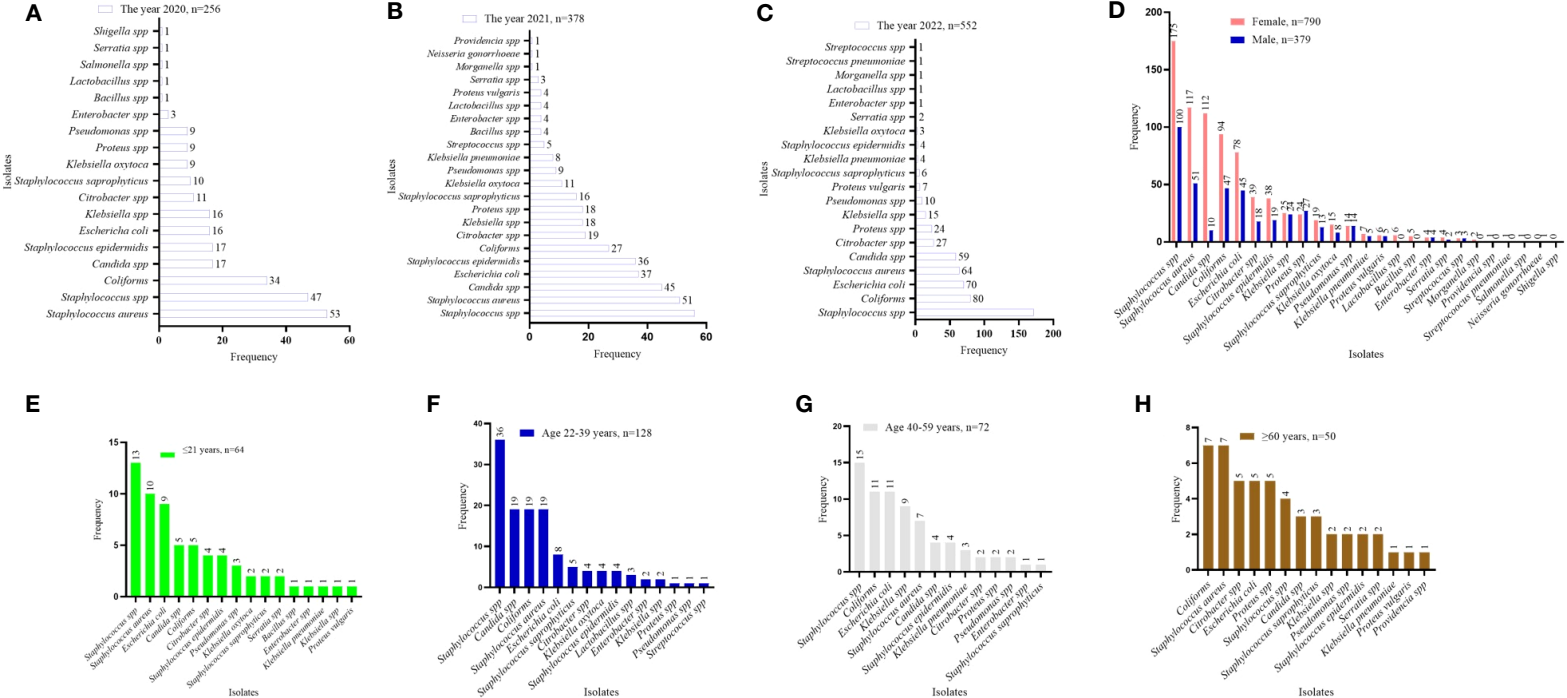
Distribution of isolates by year, sex, and age. ***(*A–C)** isolates identified by year. **(D)** distribution of isolates by sex. **(E–H)** isolate distribution per age category. The values displayed are absolute counts.

The numbers, but not the diversity, of isolates, from 2020 to 2022, were on a positive trajectory [*n* = 256 in 2020; *n* = 378 in 2021; and *n* = 552 in 2022; *N* = 1,186]. Over the 3-year period, the isolates were predominantly Staphylococcus spp., coliforms, Candida spp., and E. coli (**Figures 2A–C**). In terms of sex, the isolates were frequently from female patients, except in the case of Proteus spp. (**Figure 2D**). In terms of age, while Staphylococcus spp. was more common in the younger patients, both coliforms and Staphylococcus spp. were dominant among specimens from those patients aged ≥ 60 years. Isolate frequency per age group was 64 in those aged ≤ 21 years, 128 in 22- to 39-year-olds, 72 in 40- to 59-year-olds, and 50 in ≥ 60-year-olds (*N* = 314; **Figures 2E–H****).**

### AMR pattern observed per the WHO’s ACCESS antibiotics group

**Figure 3:**
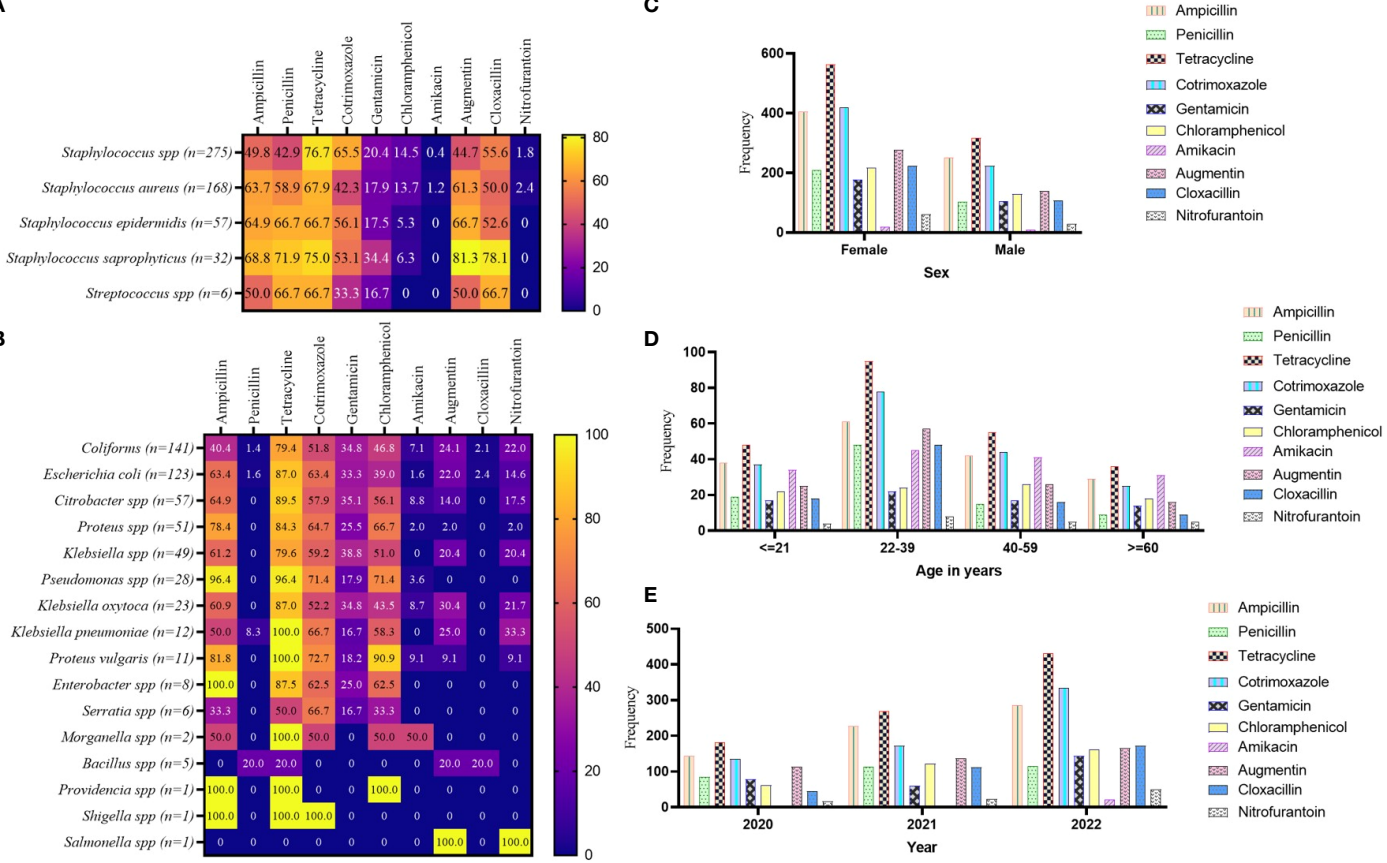
Pattern of AMR among the WHO’s ACCESS antibiotics group. **(A, B)** a heatmap comparing the antibiotic resistance pattern per Gram-positive **(A)** and Gram-negative **(B)** isolates; the values displayed are in percentages. **(C–E)** the AMR pattern by sex, age, and year of investigation, respectively; the values displayed are absolute.

Among the GP isolates assessed against the WHO’s ACCESS antibiotic group, tetracycline showed a relatively high level of non-responsiveness, this was particularly observed in *Staphylococcus* spp. (76.6%) and *Staphylococcus saprophyticus* (75.0%). *S. saprophyticus* also showed high levels of resistance to augmentin (81.3%) and cloxacillin (78.1%). The antibiotics that showed comparatively high responsiveness to the GP isolates were nitrofurantoin (ranging from 0.0% to 2.3%) and amikacin (ranging from 0.0% to 1.2%), then chloramphenicol and gentamicin (**Figure 3A**). Similarly, among the GN isolates, tetracycline non-responsiveness ranges from 20.0% (for *Bacillus* spp.) to 100% in *Klebsiella*, *Proteus vulgaris*, *Morganella*, *Providencia*, and *Shigella* species. However, the GN isolates exhibited relatively high levels of susceptibility to amikacin, augmentin, and nitrofurantoin (**Figure 3B**). In terms of stratification based on sex, the AMR observed was similar in both females and males. Comparatively, higher numbers of resistant isolates were seen in females than in male (**Figure 3C**). The pattern of AMR among the WHO’s ACCESS antibiotics groups was identical for age categories. However, isolates from patients aged 22–39 years showed the highest AMR, whereas those from patients aged ≥ 60 years demonstrated the lowest AMR (**Figure 3D**). Assessing the AMR levels per the year of the laboratory test for culture and sensitivity assay, the data showed that the incidence of AMR was on a positive trajectory from 2020 to 2022 (**Figure 3E**), which is in concordance with the sample volume received by the laboratory.

### AMR pattern observed per the WHO’s WATCH antibiotics group

**Figure 4:**
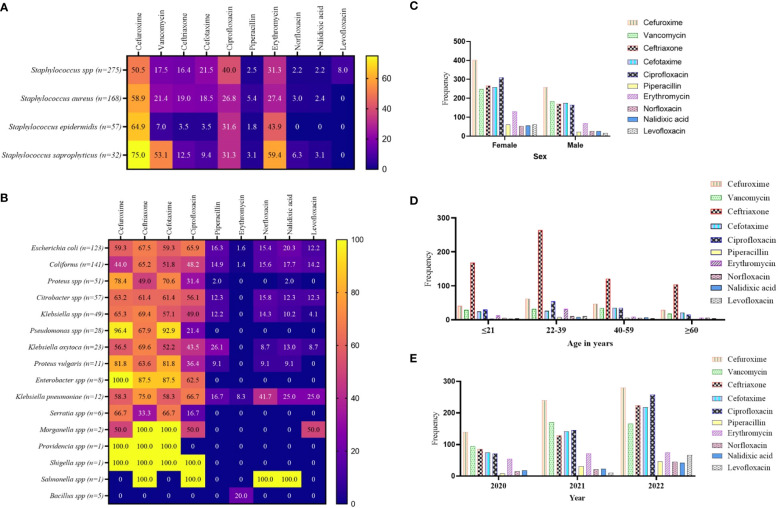
Pattern of AMR among the WHO’s WATCH antibiotics group. **(A, B)** a heatmap comparing the antibiotic resistance pattern per Gram-positive **(A)** and Gram-negative **(B)** isolates; the values are displayed in percentages. **(C–E)** the AMR pattern by sex, age, and year of investigation respectively; the values displayed are absolute.

From the culture and sensitivity test results, the WHO’s WATCH antibiotics were extracted and analyzed for patterns of AMR. For the GP isolates, the study revealed relatively high levels of effectiveness by the WHO’s WATCH antibiotics. However, cefuroxime exhibited relatively high levels of non-responsiveness to *S. saprophyticus* (75.0%), *Staphylococcus epidermidis* (64.9%), *S. aureus* (58.9%), and *Staphylococcus* spp. (50.0%). In addition, the GP isolates showed 31.3% to 40.4% resistance to ciprofloxacin, and 27.4% to 59.4% resistance to erythromycin. The antibiotics norfloxacin, piperacillin, nalidixic acid, and levofloxacin remained significantly potent against the GP isolates (**Figure 4A**). In a similar fashion, the antibiotics cefuroxime, ceftriaxone, cefotaxime, and ciprofloxacin showed relatively high levels of non-responsiveness to the GN isolates, particularly *Pseudomonas* spp. (ranging from 21.4% to 96.4%), *Proteus vulgaris* (ranging from 36.4% to 81.8%), and *Enterobacter* spp. (ranging from 62.5% to 100%). *Morganella*, *Providencia*, *Shigella*, and *Salmonella* species were predominantly 100% non-responsive to these antibiotics. However, piperacillin, erythromycin, norfloxacin, nalidixic acid, and levofloxacin exhibited varied degrees of effectiveness against the GN isolates (**Figure 4B**). Per the stratification based on a patient’s sex to the WHO’s WATCH antibiotics, resistance patterns showed marginal variations, especially with ciprofloxacin (**Figure 4C**). Comparing the WATCH antibiotics to a patient’s age, the most frequent non-responsiveness in all age groups was ceftriaxone and the least was levofloxacin (**Figure 4D**). The study also exposed an increasing pattern of AMR incidence in this group of antibiotics as the years progressed (**Figure 4E**).

### AMR pattern observed per the WHO’s RESERVE antibiotics group

**Figure 5:**
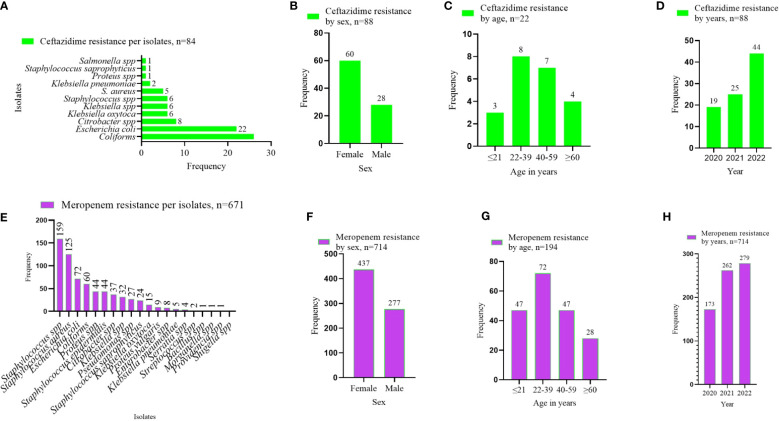
AMR pattern observed per the WHO’s RESERVE antibiotics group. **(A–D)** shows the ceftazidime resistance pattern by isolates, sex of patients, age category of patients, and year of investigation. **(E–H)** displays the meropenem resistance pattern by isolates, sex of patients, age category of patients, and year of investigation. The values displayed are absolute.

In stratifying the antibiotics tested to the WHO’s AWaRe classification, two antibiotics were identified under the WHO’s RESERVE: ceftazidime and meropenem. Resistance to ceftazidime was commonly associated with the GN isolates, that is, coliforms, *E. coli*, *Klebsiella*, and *Citrobacter* species (**Figure 5A**). The number of resistant isolates to ceftazidime was more than twice as high in females (68.2%, *n* = 60) than in males (31.8%, *n* = 28) (**Figure 5B**). More than half of the ceftazidime-resistant cases were recorded in patients aged 22–59 years (68.2%, 15/22) (**Figure 5C**), whereas the incidence of resistance more than doubled in 2022 compared with 2020 (**Figure 5D**). The resistance to meropenem was common in *Staphylococcus* spp., *E. coli*, coliforms, and *Proteus* spp. (**Figure 5E**). Female patients recorded more meropenem-resistant cases than males (**Figure 5F**), and such resistance peaked among patients in the age category 22–39 years (**Figure 5G**), with an increasing number of cases of resistance over the period (**Figure 5H**).

### Multidrug resistance isolates as defined by ECDC-MDR

**Figure 6:**
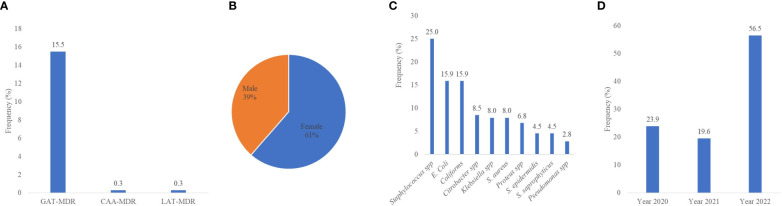
Multidrug resistance isolates, as defined by the ECDC-MDR classification. MDR, multidrug resistance; GAT-MDR, gentamycin, ampicillin, and tetracycline MDR; CAA-MDR, ciprofloxacin, amikacin, and ampicillin MDR; LAT-MDR, levofloxacin, amikacin, and tetracycline MDR.

The data were applied to the definition of multidrug resistance, as described by the ECDC. The ECDC-MDR was categorized into three groups based on the permutations of the most dominant antibiotics used in this study. These groups were (1) GAT-MDR (gentamycin, ampicillin, and tetracycline MDR); (2) CAA-MDR (ciprofloxacin, amikacin, and ampicillin MDR); and (3) LAT-MDR (levofloxacin, amikacin, and tetracycline MDR). Out of the 1,187 isolates, 15.5% (184) were GAT-MDR, and 0.3% each for CAA-MDR and LAT-MDR isolates (**Figure 6A**). Since most of the isolates were GAT-MDR, an expanded analysis was performed on these groups of isolates. Considering the patients’ sex, 61% (112/184) of GAT-MDR isolates were from females (**Figure 6B**). The most predominant GAT-MDR were *Staphylococcus* spp., *E. coli*, coliforms, and *Klebsiella* spp., whereas the least was *Pseudomonas* spp. (**Figure 6C**), with MDR peaking in 2022 (**Figure 6D**).

In the present study, fewer samples were received in 2020 than in 2021 and 2022. It has been reported that the occurrence of COVID-19 in 2020, which had many implications on human life, with subsequent restrictions and lockdowns, greatly affected attendance at health facilities (Ferrero et al., 2020; Moyer et al., 2021; Poyser et al., 2021). The easing of COVID-19 restrictions and the return to normal life could account for the increasing specimen numbers in 2021 and 2022. The increasing specimen numbers also corresponds to the increasing number of cases of AMR. It is believed that there were significant antibiotic abuses during the COVID-19 pandemic, and the surge in AMR post-COVID-19 could be partly due to practices in antibiotic use in the COVID-19 era (Garg, 2021). However, this assumption will need further scientific investigation.

There was an observed higher number of samples received from females than from males. Anecdotally, more females attend health facilities than males. Among pregnant mothers, it is compulsory for routine antenatal clinic visits, and this contributes to the large number of female specimens. In general, AMR was predominant in females, likewise the incidence of MDR in females (61%). Some studies have linked the predominantly high number of pathogenic bacterial isolates from females, particularly in “uropathogenic” isolates, to their susceptibility to such infections due to their physiology and anatomy (Gu et al., 2022). The majority of clinical specimens, as well as isolates, were within patients aged 22 years to 39 years, and this reflects the youthful nature of the population, particularly among women of reproductive age. However, only 25.7% (819) of the available patient data included their age.

According to the WHO’s ACCESS antibiotic group, the study data exhibited high levels of resistance to tetracycline, cotrimoxazole, cloxacillin, and penicillin, which ranged from 22.2% to 100%, with relatively low levels of resistance observed among the GP isolates when compared with the GN isolates. Antibiotics that showed comparatively high levels of responsiveness to the GP isolates were nitrofurantoin (0.0% to 2.3%), amikacin (0.0% to 1.2%), then chloramphenicol and gentamicin. In Ghana, other studies have shown high rates of prevalence of resistance to tetracycline (82%), cotrimoxazole (73%), ampicillin (76%), and chloramphenicol (75%). An ampicillin, tetracycline, chloramphenicol, and cotrimoxazole combination was also found to have multidrug resistance, with ceftriaxone (6.3%), ciprofloxacin (11%) and amikacin (9.9%) having lower resistance percentages (Newman et al., 2011). This observation suggests that the current prevalence of AMR per the WHO ACCESS classification agrees with previous studies (Jia et al., 2023; Vicar et al., 2023).

Analyzing for AMR with regard to the WHO’s WATCH antibiotics, relatively high levels of resistance were observed for cefuroxime, especially in *Staphylococcus* species, ranging from 50.0% to 75.0%. The resistance to ciprofloxacin ranges between 31.3% and 40.4%, and between 27.4% and 59.4% for erythromycin. Interestingly, most of the GN isolates, particularly *Proteus*, *Pseudomonas*, *Enterobacter*, *Salmonella*, and *Shigella* species demonstrated high levels of resistance to cefuroxime, ceftriaxone, cefotaxime, and ciprofloxacin. Other studies showed high levels of resistance to trimethoprim/sulfamethoxazole (84.5%), cefuroxime (79%), and cefotaxime (71.3%) (Obeng-Nkrumah et al., 2016; Agyepong et al., 2018). The AMR among the two WHO RESERVE antibiotics used was 22.3% (159/714) for meropenem and 35.2% (31/88) for ceftazidime. The study revealed relatively low usage of this group of antibiotics, and contradicts the AMR prevalence of similar antibiotics in previous studies; for example, ertapenem (1.5%), meropenem (3%), and amikacin (11%) (Obeng-Nkrumah et al., 2016; Agyepong et al., 2018).

By applying the ECDC-MDR definition to the antibiotics used in this study, *Staphylococcus* spp. (25.0%) were the isolates leading in MDR, followed by *E. coli* (15.9%). However, grouping the isolates into GN and GP, 58.0% of the isolates were GN and 42% GP. The overall MDR of 15.5% observed in this study, per the ECDC definition, contradicts previous studies on AMR which report high levels of MDR, ranging from 28% to 70% (Nkansa-Gyamfi et al., 2019; Alemayehu, 2021). Possibly, this is due to the lack of clear definitions based on national data (Yevutsey et al., 2017). When we applied the data to other definitions of MDR, such as the German KRINKO definitions and the University Hospital Zurich classification (Wolfensberger et al., 2019; Wolfensberger et al., 2019), no MDR was identified (data not shown), which is contrary to a previously published report in Ghana (Inusah et al., 2021). Possibly, this is due to the limited number of antibiotics tested for resistance. This suggests the need for an expanded AMR study to inform the definition of MDR, particularly in Ghana.

In conclusion, this study identified increasing numbers of cases of AMR, with a corresponding increase in the number of MDR isolates, as defined by the ECDC. For the first time in Ghana, we applied the WHO’s AWaRe classification of antibiotics to AMR. The results suggest diverse resistance patterns and a limiting degree of resistance moving from the ACCESS group to the RESERVE group. The ECDC-MDR classification revealed a prevalence of 15.5%. Both AMR and MDR were significantly higher in females than in males. In terms of species, *Staphylococcus* spp. was more frequently associated with both AMR and MDR. However, by Gram classification of the MDR isolates, 58.0% of them were GN, and 48.0% were GP. The major GN isolates which were MDR are *E. coli*, coliforms, *Citrobacter*, *Klebsiella*, *Proteus*, and *Pseudomonas* species, whereas the GP isolates were unspecified *Staphylococcus* species, and particularly *S. aureus*, *S. epidermidis*, and *S. saprophyticus*. The limitations identified were incomplete data and inconclusive species identification of a significant proportion of the isolates. There is therefore the need for a policy decision to enhance AMR and MDR screening for optimum clinical benefit.

## Data availability statement

The data analyzed in this study is subject to the following licenses/restrictions: The dataset for this study is available from the corresponding author, and will be made available upon reasonable request. The corresponding author has full access to all of the data in this study and takes complete responsibility for the integrity of the data and the accuracy of the data analysis. Requests to access these datasets should be directed to WW, wwalana@uds.edu.gh.

## Ethics statement

The studies involving humans were approved by the Institutional Review Board of the University for Development Studies (UDS/RB/032/23). The studies were conducted in accordance with the local legislation and institutional requirements. Written informed consent for participation was not required from the participants or the participants’ legal guardians/next of kin in accordance with the national legislation and institutional requirements.

## Author contributions

WW: Conceptualization, Data curation, Formal Analysis, Writing – original draft. EV: Data curation, Formal Analysis, Writing – review & editing. EK: Data curation, Formal Analysis, Writing – review & editing. FS: Data curation, Writing – review & editing. IY: Data curation, Writing – review & editing. EF: Data curation, Writing – review & editing. SA: Data curation, Writing – review & editing. JZ: Conceptualization, Writing – review & editing.
